# Intracellular Transposition and Capture of Mobile Genetic Elements following Intercellular Conjugation of Multidrug Resistance Conjugative Plasmids from Clinical *Enterobacteriaceae* Isolates

**DOI:** 10.1128/spectrum.02140-21

**Published:** 2022-01-19

**Authors:** Supathep Tansirichaiya, Richard N. Goodman, Xinyu Guo, Issra Bulgasim, Ørjan Samuelsen, Mohammed Al-Haroni, Adam P. Roberts

**Affiliations:** a Department of Microbiology, Faculty of Medicine Siriraj Hospital, Mahidol University, Bangkok, Thailand; b Department of Clinical Dentistry, Faculty of Health Sciences, UiT the Arctic University of Norway, Tromsø, Norway; c Centre for New Antimicrobial Strategies, UiT the Arctic University of Norway, Tromsø, Norway; d Department of Tropical Disease Biology, Liverpool School of Tropical Medicine, Liverpool, United Kingdom; e Department of Pharmacy, UiT the Arctic University of Norway, Tromsø, Norway; f Norwegian National Advisory Unit on Detection of Antimicrobial Resistance, Department of Microbiology and Infection Control, University Hospital of North Norway, Tromsø, Norway; Universidad de Buenos Aires, Facultad de Farmacia y Bioquímica

**Keywords:** entrapment vector, insertion sequence, antimicrobial resistance, transposon, *bla*_NDM-1_

## Abstract

Mobile genetic elements (MGEs) are often associated with antimicrobial resistance genes (ARGs). They are responsible for intracellular transposition between different replicons and intercellular conjugation and are therefore important agents of ARG dissemination. Detection and characterization of functional MGEs, especially in clinical isolates, would increase our understanding of the underlying pathways of transposition and recombination and allow us to determine interventional strategies to interrupt this process. Entrapment vectors can be used to capture active MGEs, as they contain a positive selection genetic system conferring a selectable phenotype upon the insertion of an MGE within certain regions of that system. Previously, we developed the pBACpAK entrapment vector that results in a tetracycline-resistant phenotype when MGEs translocate and disrupt the *cI* repressor gene. We have previously used pBACpAK to capture MGEs in clinical Escherichia coli isolates following transformation with pBACpAK. In this study, we aimed to extend the utilization of pBACpAK to other bacterial taxa. We utilized an MGE-free recipient E. coli strain containing pBACpAK to capture MGEs on conjugative, ARG-containing plasmids following conjugation from clinical *Enterobacteriaceae* donors. Following the conjugative transfer of multiple conjugative plasmids and screening for tetracycline resistance in these transconjugants, we captured several insertion sequence (IS) elements and novel transposons (Tn*7350* and Tn*7351*) and detected the *de novo* formation of novel putative composite transposons where the pBACpAK-located *tet*(A) is flanked by IS*Kpn25* from the transferred conjugative plasmid, as well as the IS*Kpn14*-mediated integration of an entire 119-kb, *bla*_NDM-1_-containing conjugative plasmid from Klebsiella pneumoniae.

**IMPORTANCE** By analyzing transposition activity within our MGE-free recipient, we can gain insights into the interaction and evolution of multidrug resistance-conferring MGEs following conjugation, including the movement of multiple ISs, the formation of composite transposons, and cointegration and/or recombination between different replicons in the same cell. This combination of recipient and entrapment vector will allow fine-scale experimental studies of factors affecting intracellular transposition and MGE formation in and from ARG-encoding MGEs from multiple species of clinically relevant *Enterobacteriaceae*.

## INTRODUCTION

Antimicrobial resistance (AMR) is a major global public health problem and is likely to get worse without the rapid development of new antibiotics and additional therapeutic options. Every use of antimicrobials provides a selective pressure for the evolution of AMR and associated mobile genetic elements (MGEs).

MGEs, such as conjugative plasmids and integrative and conjugative elements (ICEs), are responsible for the dissemination of antimicrobial resistance genes (ARGs) among bacteria, and they often contain multiple ARGs. ARGs are mobilized onto conjugative MGEs via the activity of smaller MGEs, including insertion sequences (ISs), that are capable of intracellular transposition. Transposons containing ARGs against last-resort antibiotics have been found on different plasmids in different bacterial species. For example, *bla*_NDM-1_, conferring carbapenem resistance, and *mcr-1*, conferring colistin resistance, were found on IS*Aba125-* and IS*Apl1*-based composite transposons like Tn*125* and Tn*6330*, respectively. Both composite transposons have been found on multiple plasmids in different bacterial species (1–5).

MGEs are usually identified through the phenotypic changes conferred by the accessory genes, including ARGs, or changes caused by insertions of MGEs that result in the activation/inactivation of other genes. For example, insertions of IS*26*, IS*5*, IS*903*, and IS*1* into the *ompK36* porin gene and insertion of the IS*Ecp1*-*bla*_OXA-181_ transposon into the *mgrB* gene were shown to result in carbapenem and colistin resistance, respectively, in Klebsiella pneumoniae (6–8). Bioinformatic analysis can also identify MGEs by comparative genomics of whole-genome sequencing (WGS) data and by interrogating WGS data with available databases of MGEs (9, 10); however, this approach can rarely give information on the transposition activity of these MGEs. Contextualization of AMR genes on MGEs from short-read sequencing data is also notoriously difficult (11).

**FIG 1:**
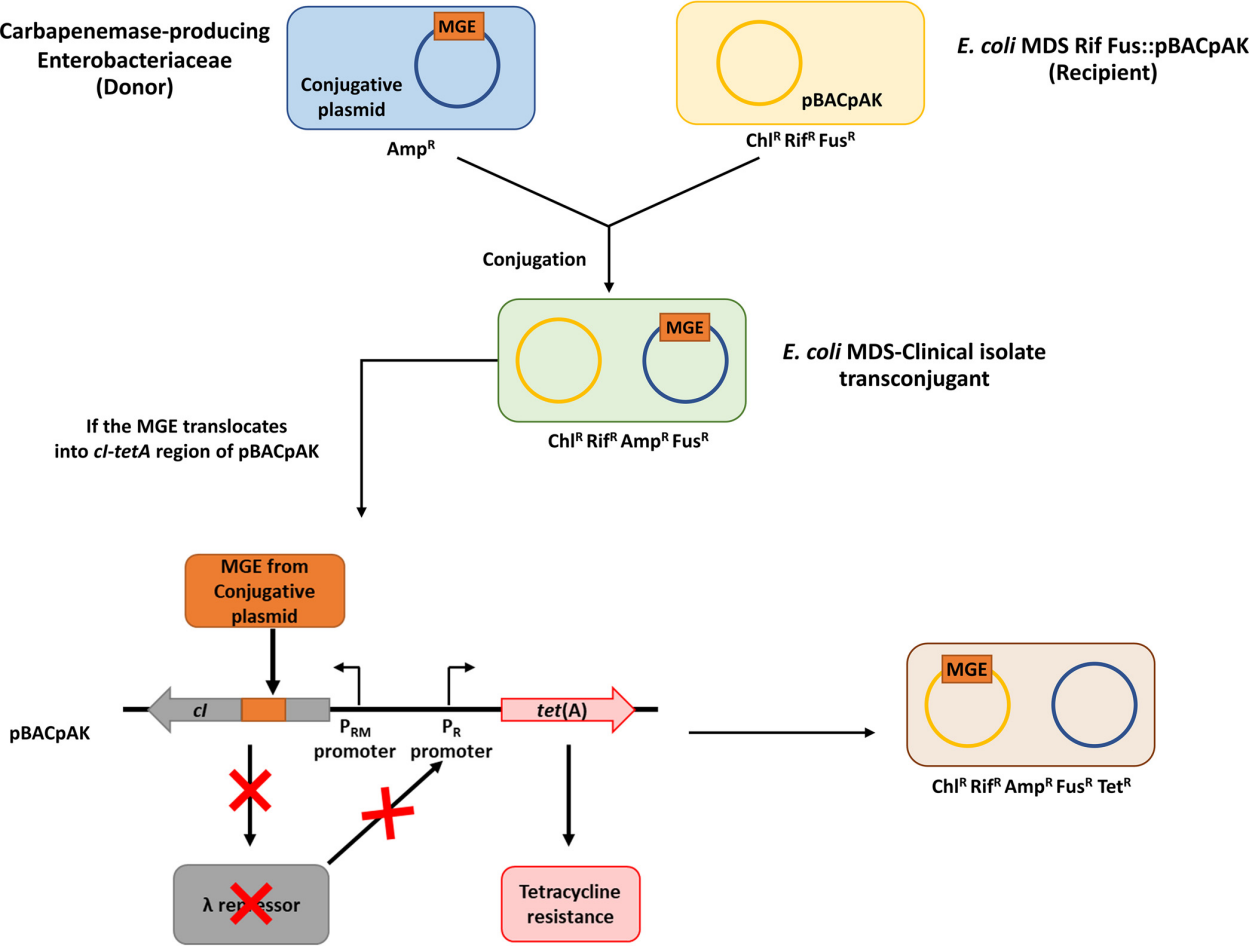
Capture of MGEs from conjugative plasmids by using pBACpAK. The conjugative plasmid from carbapenemase-producing *Enterobacteriaceae* clinical strains were transferred to an E. coli MDS Rif Fus::pBACpAK recipient strain through conjugation. Transconjugants were selected on LB agar supplemented with chloramphenicol, rifampicin, ampicillin, and fusidic acid. If the MGE, located on the conjugative plasmid, translocated into the *cI* gene on pBACpAK, it would disrupt the expression of the λ repressor, conferring tetracycline resistance due to the derepression of the P_R_ promoter. Clones with an insertion of MGEs therefore can be selected on tetracycline-containing agar. The gray and red arrowed boxes represent *cI* and *tet*(A) genes, respectively, which point in the direction of transcription. MGE, λ repressors, and tetracycline resistance protein are shown as orange, gray, and red rectangles. The blue, yellow, green, and brown rectangles represent donor, recipient, transconjugant, and tetracycline-resistant transconjugant cells, respectively. Chl^r^, chloramphenicol resistance; Rif^r^, rifampicin resistance; Amp^r^, ampicillin resistance; Fus^r^, fusidic acid resistance; Tet^r^, tetracycline resistance.

Another approach is to use entrapment vectors to capture MGEs based solely on their transposition activity. Entrapment vectors contain a genetic system that will confer a selective phenotype when MGEs transpose into a defined region of DNA (12–14). Previously, we developed a single-copy-number entrapment vector called pBACpAK and demonstrated that it can detect the insertion of MGEs in both laboratory and clinical Escherichia coli isolates (15, 16). pBACpAK contains a *cI*-*te*(A) gene system in which the λ repressor (encoded by *cI*) constitutively inhibits the expression of the *tet*(A) gene by binding to the P_RM_ promoter, blocking the expression of *tet*(A) (17). When an MGE inserts into the *cI* gene, the expression of the repressor is interrupted, leading to the expression of *tet*(A) and a tetracycline resistance phenotype (Fig. 1). Several IS elements and a novel translocatable unit (TU) carrying a functional trimethoprim resistance gene, *dfrA8*, were captured by using the pBACpAK entrapment vector previously (15).

In this study, we used pBACpAK to identify active MGEs from conjugative plasmids that had transferred via conjugation to a transposon-free, differentially resistant recipient E. coli strain from carbapenemase-producing clinical *Enterobacteriaceae* donor strains (Fig. 1). Multiple novel MGEs were detected from the screening of tetracycline-resistant transconjugants, giving insights into the interaction and evolution of MGEs carrying ARGs.

## RESULTS

### Characterization of donor and recipient strains.

Based on bioinformatic analysis of the WGS data of 59 carbapenemase-producing *Enterobacteriaceae* clinical strains, 8 clinical isolates (7 K. pneumoniae and 1 E. coli) were selected as donors in this study, as summarized in Table 1, with the raw data shown in Table S2 in the supplemental material. Their resistance phenotypes against rifampicin (Rif) and fusidic acid (Fus) were subsequently determined to make sure that both antibiotics could be used for selection of transconjugants following a filter-mating experiment. All 8 clinical strains showed no growth on Luria-Bertani (LB) agar supplemented with rifampicin and fusidic acid (Table 1).

**TABLE 1:** The details of carbapenemase-producing *Enterobacteriaceae* clinical strains

Species	Isolate	No. of:		β-Lactamase(s) associated with plasmid-derived contigs^*b*^	Resistance determinant(s)^*b*^^,^^*c*^		Resistance phenotype^*c*^		
		Plasmids^*a*^	MGEs associated with plasmid-derived contigs^*b*^		Chl	Tet	Chl	Tet	Rif and Fus
K. pneumoniae	K57-33	6	10	*bla*_OXA-9_, *bla*_TEM-1A_, *bla*_KPC-2_	No	No	S	S	S
K. pneumoniae	K68-18	6	6	*bla*_VIM-27_	No	No	R	S	S
K. pneumoniae	K46-62	2	6	*bla*_SHV-12_, *bla*_TEM-1B_, *bla*_VIM-1_	No	No	S	S	S
K. pneumoniae	50825040	4	11	*bla*_CTX-M-15_, *bla*_OXA-9_, *bla*_TEM-1B_, *bla*_NDM-1_	No	No	S	S	S
K. pneumoniae	50877064	1	4	*bla*_CMY-6_, *bla*_NDM-1_	No	No	S	S	S
K. pneumoniae	50675619	5	8	*bla*_NDM-7_, *bla*_OXA-1_, *bla*_CTX-M-15_, *bla*_TEM-1B_	*catB3* (P)	No	S	S	S
K. pneumoniae	50627996	3	6	*bla*_CMY-6_, *bla*_NDM-1_, *bla*_CTX-M-15_	*catA1* (P), *catB3* (C)	No	R	R	S
E. coli	50676002	4	5	*bla*_CMY-6_, *bla*_OXA-1_, *bla*_NDM-1_	*catB3* (P)	*tet*(A) (C)	S	R	S

a The number of plasmids was predicted by using PlasmidFinder (49).

b MGEs and ARGs were analyzed from WGS data by using Mobile Element Finder and ResFinder, respectively (10, 47). mlplasmid was used to predict that the contigs containing each MGE and ARG were likely to be either chromosome-derived (C) or plasmid-derived (P) DNA (48).

c Chl, chloramphenicol; Tet, tetracycline; Rif, rifampicin; Fus, fusidic acid; S, susceptible; R, resistance.

pBACpAK was electroporated into E. coli strain MDS, which was subsequently sequentially evolved to rifampicin and then fusidic acid resistance. The strain was then screened on LB agar containing tetracycline to check for the rate of mutations in the *cI* gene. No tetracycline-resistant colonies were found on any plates from three replicates after 72 h of incubation.

Developing the E. coli MDS::pBACpAK strain into rifampicin and fusidic acid resistance was done to use the resistant phenotypes as selective markers for recipient strains, reducing the chance for false-positive transconjugants due to spontaneous mutations in the donor strains. E. coli MDS::pBACpAK with rifampicin and fusidic acid resistance was selected and denoted E. coli MDS Rif Fus::pBACpAK. E. coli MDS Rif Fus::pBACpAK was shown to have point mutations (underlined) in the *rpoB* (D516G [a mutation of D to G at position 516]; GAC→GGC) and *fusA* (L466F; CTC→TTC) genes, which are known to confer resistance to rifampicin and fusidic acid, respectively (18, 19).

### Transfer of conjugative plasmids from clinical isolates to E. coli MDS Rif Fus::pBACpAK.

Filter mating between clinical isolates and E. coli MDS Rif Fus::pBACpAK was carried out, and transconjugants were selected on LB agar supplemented with chloramphenicol, rifampicin, ampicillin, and fusidic acid (LB CRAF agar). Several transconjugant colonies were found from all mating pairs. The donor-only group showed no growth on LB agar supplemented with chloramphenicol, rifampicin, and fusidic acid, while the recipient-only group showed no growth on LB ampicillin plates. Both control groups also showed no growth on any LB CRAF agar plates. The *cI-tet*(A) fragments were successfully amplified from all mating pairs except the K. pneumoniae strain K68-18 and E. coli MDS Rif Fus::pBACpAK pair, which verified that transconjugants from 7 donors contained conjugative plasmids (conferring an ampicillin resistance phenotype) and the pBACpAK entrapment vector.

### Screening for tetracycline resistance transconjugants.

**FIG 2:**
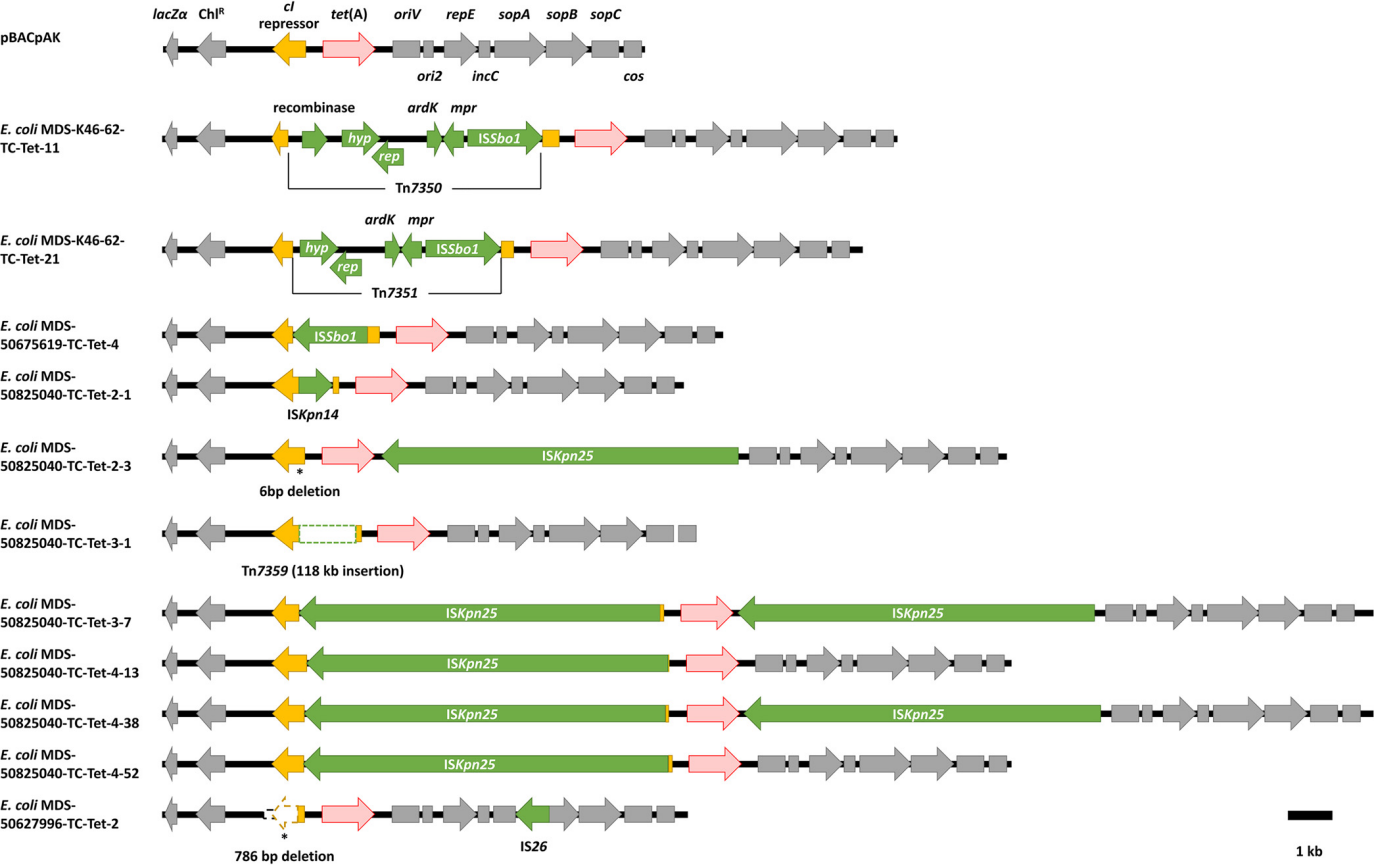
The structures of MGEs captured by pBACpAK in tetracycline-resistant transconjugants. The red, yellow, green, and grey arrowed boxes represent *tet*(A), *cI*, MGEs, and other genes, respectively. The green dashed box represents an insertion of Tn*7359*, shown in Fig. 4.

All 7 transconjugants from 7 donors were grown on agar plates containing LB CRAF agar plus tetracycline (LB CRAFT agar) to select for colonies with a tetracycline resistance phenotype. Characterizing each tetracycline-resistant clone identified 11 clones with insertion of MGEs on pBACpAK (Table 2). Four of them (E. coli MDS-K46-62-TC-Tet-11, E. coli MDS-K46-62-TC-Tet-21, E. coli MDS-50675619-TC-Tet-4, and E. coli MDS-50825040-TC-Tet-2-1) were characterized by sequencing their *cI*-*tet*(A) amplicons, while the rest failed to be amplified by PCR, so they were characterized by WGS. The results showed that pBACpAK captured 4 different IS elements (IS*26*, IS*Sbo1*, IS*Kpn14*, and IS*Kpn25*) and 2 novel transposons (designated Tn*7350* and Tn*7351*) (Fig. 2). We also detected a recombinant pBACpAK::p50825040 plasmid molecule. Plasmid p50825040 is a previously unnamed plasmid (20) that we transferred from the K. pneumoniae 50825040 donor.

**TABLE 2:** The details of MGEs captured by the pBACpAK entrapment vector

Strain (accession no.)	MGE/mutation	Location on pBACpAK (position)	Size (bp)	% similarity (ISFinder/NCBI)			Direct repeat
				BLASTN	BLASTX	Accession no.	
E. coli MDS-K46-62-TC strains							
Tet-11	Tn*7350*	*cI* repressor (2874)	5,812	99.12		HF955507	
	Recombinase family protein		570		99	WP_195767670	
	Hypothetical protein		849		91	SAP93263	
	RepB family plasmid replication initiator protein		720		99	WP_087871684	
	Transcriptional regulator *ardK*		342		100	ACI63129	
	Zinc metalloproteinase Mpr		465		99	CDF32051	
	IS*Sbo1*		1,709	96		CP001062	

Tet-21	Tn*7351*	*cI* repressor (2963)	4,796	99.25		HF955507	GAAC
	Hypothetical protein		849		95	SXT51837	
	RepB family plasmid replication initiator protein		720		99	WP_087871684	
	Transcriptional regulator *ardK*		342		100	ACI63129	
	Zinc metalloproteinase Mpr		465		99	CDF32051	
	IS*Sbo1*		1,709		94	CP001062	

E. coli MDS-50675619-TC-Tet-4	IS*Sbo1*	*cI* repressor (2976)	1,709	96	98	CP001062	GAAC

E. coli MDS-50825040-TC strains							
Tet-2-1	IS*Kpn14*	*cI* repressor (3127)	768	100	100	CP000649	CGGCGTTA
Tet-2-3 (SAMN21542910)	IS*Kpn25*	Between *tet(A)* and *oriV* (4976)	8,154	100	100	NC_009650	TATTTATC
	Deletion	*cI* repressor (3174–3179)	6				
Tet-3-1 (SAMN21542911)	K. pneumoniae *bla*_NDM-1_ plasmid p2	*cI* repressor (3126)	118,959	99.99		CP009115	TAACGCCG
Tet-3-7 (SAMN21542912)	IS*Kpn25*_1	*cI* repressor (3224)	8,154	100	100	NC_009650	CATTTTTC
	IS*Kpn25*_2	Between *tet(A)* and *oriV* (4980)	8,154	100	100	NC_009650	TTTTATTT
Tet-4-13 (SAMN21542913)	IS*Kpn25*	*cI* repressor (3294)	8,154	100	100	NC_009650	CTTTTTTG
Tet-4-38 (SAMN21542914)	IS*Kpn25*_1	*cI* repressor (3230)	8,154	100	100	NC_009650	AAAAAAAG
	IS*Kpn25*_2	Between *tet(A)* and *oriV* (4941)	8,154	100	100	NC_009650	AATCTTCT
Tet-4-52 (SAMN21542915)	IS*Kpn25*	*cI* repressor (3227)	8,154	100	100	NC_009650	AAAGAAAA

E. coli MDS-50627996-TC-Tet-2 (SAMN21542916)	IS*26*	*sopA* (8066)	820	100	100	X00011	CAGATCTT
	Deletion	*cI* repressor (2305–3090)	786				

Most clones showed an insertion in the *cI* repressor genes; however, E. coli MDS-50825040-TC-Tet-2-3 and E. coli MDS-50627996-TC-Tet-2 showed an IS*Kpn25* insertion between *tet*(A) and *oriV* and an IS*26* insertion in the *sopA* gene on pBACpAK, respectively. The tetracycline resistance phenotype in both clones was a result of a deletion in the *cI* repressor gene (6-bp and 786-bp deletions). E. coli MDS-50825040-TC-Tet-3-7 and E. coli MDS-50825040-TC-Tet-4-38 each had an insertion of IS*Kpn25*, one in the *cI* repressor gene and the other one between *tet*(A) and *oriV*. For clones that were analyzed by WGS, translocation of MGEs into chromosomal DNA was determined by using breseq, which showed no additional insertion within the chromosome of the recipient in any tetracycline-resistant transconjugants.

**FIG 3:**
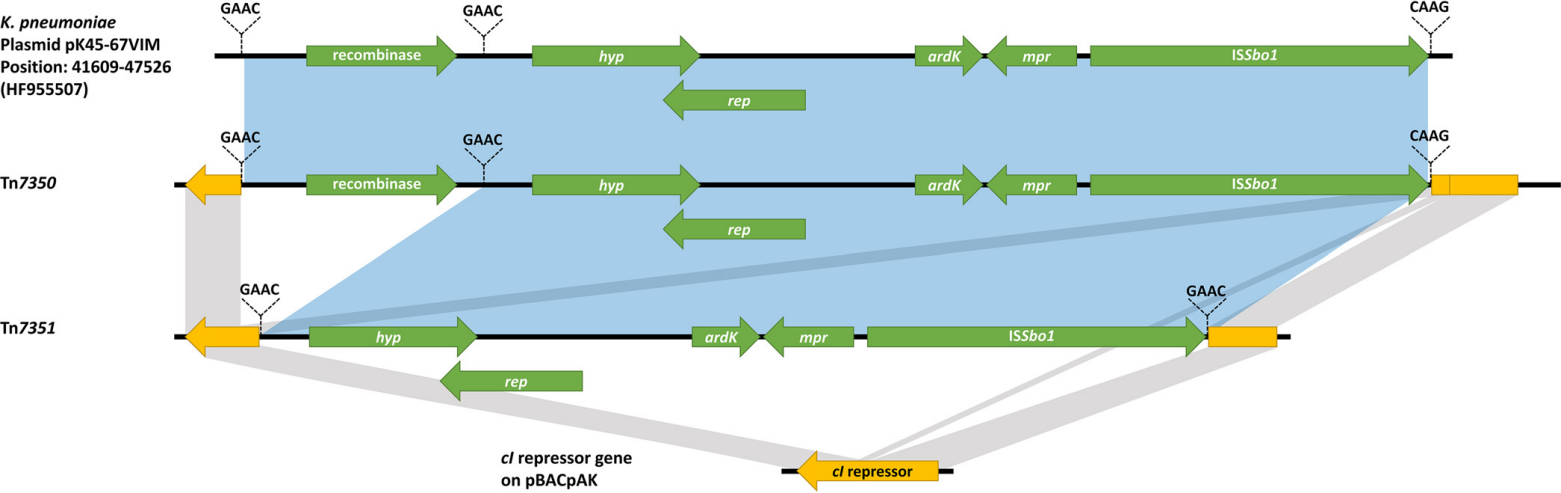
Comparison of Tn*7350* and Tn*7351* inserted in the *cI* repressor gene on the pBACpAK entrapment vector. Tn*7350* and Tn*7351* were compared to the *cI* repressor gene on pBACpAK and plasmid pK45-67VIM from K. pneumoniae (accession number HF955507). The yellow and green arrowed boxes represent the *cI* repressor gene and genes found on Tn*7350* and Tn*7351*, respectively. The identical DNA regions of the *cI* repressor gene and transposons are shown in gray and blue, respectively. The GAAC direct repeats on Tn*7350* and inverted repeats on Tn*7351* are shown and indicated with dashed lines.

Tn*7350* and Tn*7351* were identified from E. coli MDS-K46-62-TC-Tet-11 and E. coli MDS-K46-62-TC-Tet-21, respectively (Fig. 3). They were similar transposons with 99% identity to a part of plasmid pK45-67VIM found *in* K. pneumoniae (accession number HF955507) (Fig. S1A). Tn*7351* was 1,016 bp shorter than Tn*7350*, missing a recombinase (resolvase) gene. Both transposons contained an IS*Sbo1* insertion sequence and genes encoding a replication initiator protein, an ArdK transcriptional regulator, and an Mpr zinc metalloproteinase. They had different insertion sites on the pBACpAK vector (Fig. 3 and Fig. S2), and only Tn*7351* contained a 4-bp direct repeat (GAAC) (Fig. 3). The recombinase gene found only in Tn*7350* was similar to the resolvase genes from Tn*552*, Tn*917*, and Tn*2501* (accession numbers P18358, P06693, and P05823), with percent identities of 42.33%, 35.64%, and 29.10%, respectively. The region of Tn*7350* that was not present in Tn*7351* was also flanked by the direct repeat GAAC (Fig. 3).

**FIG 4:**
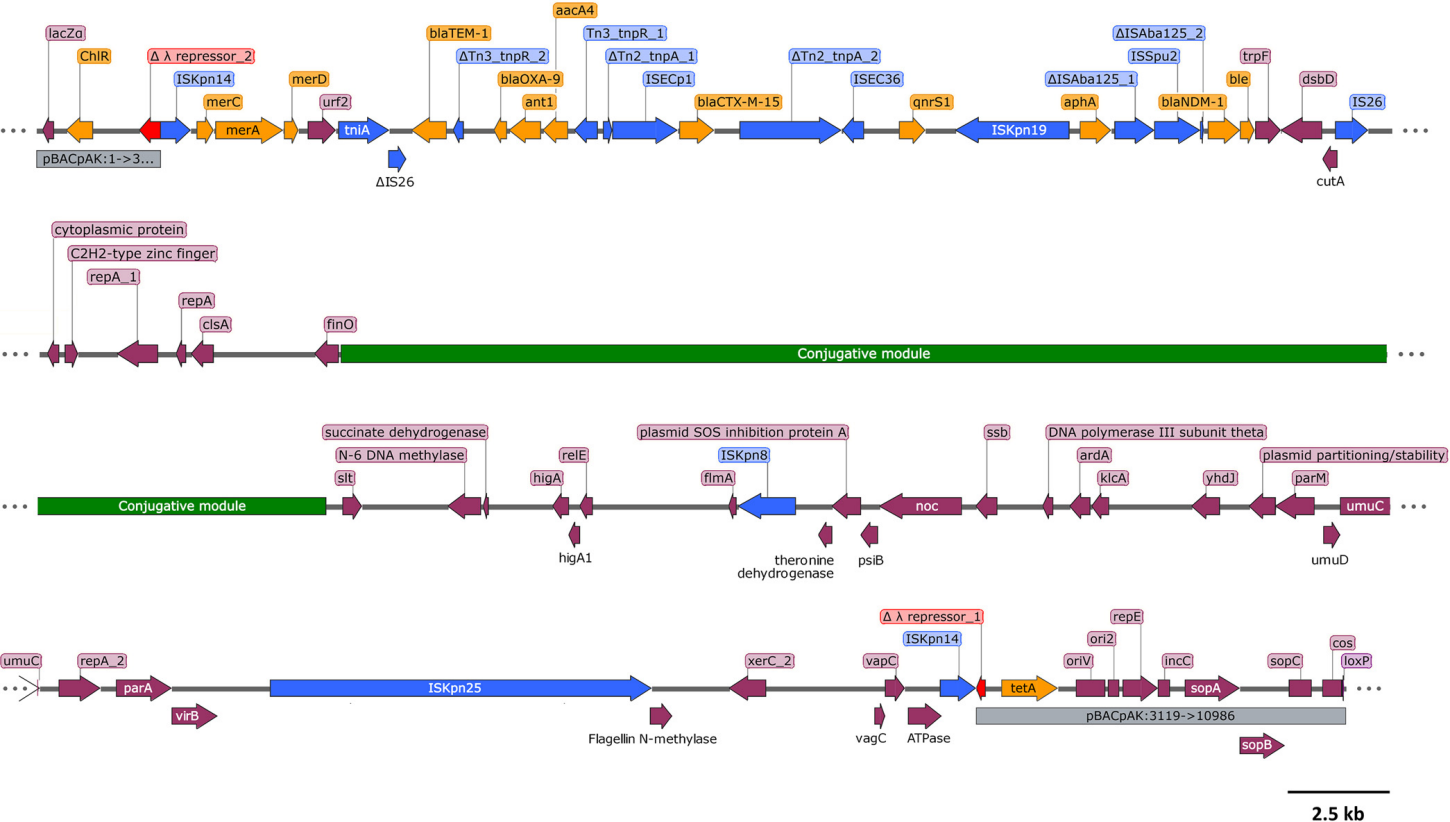
The structure of Tn*7359* captured by the pBACpAK entrapment vector from E. coli MDS-50825040-TC-Tet-3-1. The blue, yellow, red, green, and purple arrowed boxes represent MGEs, ARGs, the *cI* repressor gene, conjugative genes, and other genes, respectively. The gray boxes represent pBACpAK. Hypothetical genes were omitted from the figure. The figure was constructed using SnapGene software (Insightful Science, USA).

The insertion in E. coli MDS-50825040-TC-Tet-3-1 consisted of the p50825040 plasmid originally from the K. pneumoniae 50825040 donor. As the inserted plasmid sequence was flanked by IS*Kpn14* elements in pBACpAK, it fit the definition of a composite transposon (21) and was named Tn*7359* (Fig. 4). It was also highly similar to the *bla*_NDM-1_-containing plasmid p2 found in K. pneumoniae (accession number CP009115) (Fig. S1B) (22). Tn*7359* contained a Tn*21*-like structure (*merCAD* mercury resistance genes, *urf2*, and *tniA* genes) and multiple ARGs within an IS*26*-based pseudo-compound transposon (PCT)-like structure (23), as one of the IS*26* elements was disrupted by the *tniA* gene, a conjugative module, and a plasmid stability/replication module (Fig. 4). The IS*26* PCT-like structure contained multiple β-lactamase genes (*bla*_TEM-1_, *bla*_OXA-9_, *bla*_CTX-M-15_, and *bla*_NDM-1_), aminoglycoside resistance genes (*ant1*, *aacA4*, and *aphA*), the *ble* bleomycin resistance gene, and the *qnrS1* quinolone resistance gene. It also carried several IS elements, such as IS*Ecp1*, IS*Ec36*, IS*Spu2*, IS*Kpn19*, IS*Kpn8*, and IS*Kpn25*.

## DISCUSSION

Entrapment vectors have been used to capture MGEs in multiple bacterial species, both Gram positive and Gram negative, such as Paracoccus pantotrophus, Rhodococcus fascians, Agrobacterium tumefaciens, Corynebacterium glutamicum, and E. coli (15, 24–26). As this approach relies on the transposition activity of MGEs, it has the potential to identify new MGEs that have not been previously delineated (24, 27, 28). In our study, we have identified 2 novel transposons (Tn*7350* and Tn*7351*) in transconjugants from the conjugations between carbapenemase-producing clinical *Enterobacteriaceae* isolates and E. coli MDS Rif Fus::pBACpAK. Both of these transposons, plus the region absent in Tn*7351* compared to Tn*7350*, are flanked by GAAC inverted or direct repeats. GAAC represents the conserved target site of IS*91* insertion sequences (29, 30). IS*Sbo1*, which is present on both Tn*7350* and Tn*7351*, is a member of the IS*91* family of insertion sequences (31) and is therefore likely to be responsible for the movement of these novel transposons; however, experimental verification is still needed to confirm this.

Prior to the filter-mating experiment, we characterized each carbapenemase-producing *Enterobacteriaceae* strain by using the bioinformatic tools Mobile Element Finder, ResFinder, and PlasmidFinder to analyze their WGS data to use as criteria for a selection of donor strains. Our results showed that we detected novel MGEs with pBACpAK that were not reported by these tools, as they are not present in the databases, but it could also capture other known MGEs that were missed by these tools, such as IS*Sbo1* in K. pneumoniae 50675619, IS*Kpn25* in K. pneumoniae 50825040, and IS*26* in K. pneumoniae 50627996 (Table S2).

Previously, the pBACpAK entrapment vector was developed and used in laboratory and clinical E. coli isolates, as it was designed based on the pCC1BAC vector containing the E. coli F factor single-copy origin of replication so that entrapment of larger DNA fragments would be more stable than if we had used a high-copy-number plasmid. In this study, we proposed another approach that could extend the uses of pBACpAK to the detection of MGEs from other bacterial species through a filter-mating experiment between clinical *Enterobacteriaceae* isolates as donors and the differentially marked E. coli MDS Rif Fus::pBACpAK as a recipient. This allows the capture of MGEs located on conjugative plasmids/transposons from any bacterial species that can transfer MGEs via conjugation (or transformation) to our MGE-free E. coli recipient strain. Conjugative elements, especially from clinical isolates, tend to carry not only multiple ARGs but also smaller MGEs; it has been shown, for example, that bacterial plasmids tend to contain a significantly higher number of IS elements than their chromosomal DNA (32).

This approach also extends the uses of pBACpAK in terms of resistance phenotype, since pBACpAK uses the chloramphenicol resistance gene as a selective marker for the vector and a tetracycline resistance phenotype to screen for clones with MGE insertion. Therefore, it cannot be used directly with E. coli strains with either a chloramphenicol or tetracycline resistance phenotype. As only resistance genes associated with conjugative elements will be transferred to the recipient in filter mating, it will reduce the background resistance phenotypes from the clinical isolates. This was shown in our study where K. pneumoniae 50627996 and E. coli 50676002 had the tetracycline resistance phenotype, but the transconjugants from both clinical isolates were susceptible to tetracycline, allowing us to screen for MGEs on other conjugative elements from both strains.

The conjugation and subsequent detection of MGE movement in transconjugants demonstrate both how MGEs like IS elements and Tns can translocate from one bacterial cell to another with the help of conjugative elements and the consequences of rapid dissemination to other replicons in the recipient cell. The translocation of IS elements can have direct consequences for resistance to their host. IS*Kpn26* has recently been shown to insert into *acrR*, leading to inactivation of the AcrAB-TolC multidrug efflux pump and resistance to tigecycline in carbapenemase (KPC-2)-producing ST11 K. pneumoniae isolates from Chinese hospitals (33). IS*Kpn14* and IS*Kpn25* have previously been shown in several studies to be associated with colistin resistance through an insertion that disrupts the expression of the *mgrB* regulator gene, which results in overexpression of PhoPQ, activating the *pmrHFIJKLM* operon and modification of lipopolysaccharide, a drug target of colistin (34–38). While the translocation of ISs in our entrapment vector is also detected by interrupting a gene (*cI*) leading to a tetracycline resistance phenotype, the translocation of MGEs following conjugation can also result in the formation of new composite transposons and variations of known MGEs containing antibiotic resistance accessory genes. In our study, we observed the formation of two independently derived putative novel composite transposons containing the pBACpAK-located *tet*(A) tetracycline resistance gene flanked by copies of IS*Kpn*25. The *tet*(A) in these clones may have the potential to be disseminated as a composite transposon. It could also move through an intermediate circular structure containing one copy of IS*Kpn25* and the *tet*(A) gene, such as translocatable units (mainly reported in IS2*6*-family PCTs) and unconventional circularized structures (UCSs) (39, 40). However, the estimation of copy numbers of the *tet*(A) resistance gene in E. coli MDS-50825040-TC-Tet-3-7 and E. coli MDS-50825040-TC-Tet 4-38 (Table S3) showed that they had the same copy number as the chloramphenicol resistance gene on pBACpAK, suggesting that it was unlikely that *tet*(A) was being mobilized, and potentially amplified, from pBACpAK at detectable levels in the bacterial population analyzed; however, planned evolutionary studies will reveal if this gene is able to be acquired by the larger conjugative plasmid.

**FIG 5:**
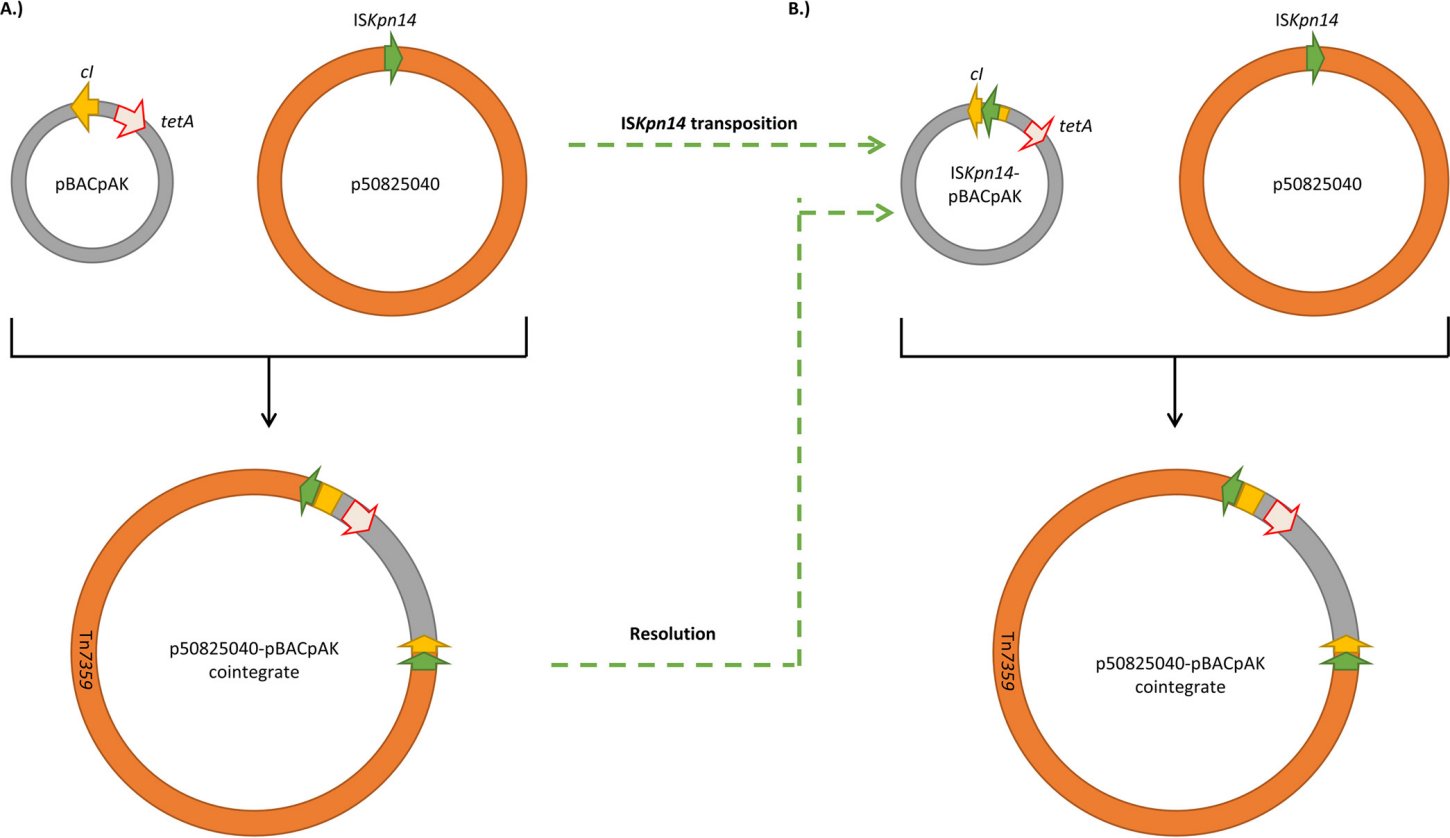
An insertion of Tn*7359* into pBACpAK. (A) Tn*7359* could form from a replicative transposition (as detailed in Biel and Berg [41]) where the cointegrate did not resolve, so it combined p50825040 with pBACpAK. (B) It could also form through targeted conservative transposition or homologous recombination between the IS*Kpn14-*containing pBACpAK and p50825040. The IS*Kpn14-*containing pBACpAK could form either through the transposition of IS*Kpn14* into the *cI* repressor gene or the resolution of the p50825040-pBACpAK cointegrate, indicated by the green dashed-line arrows. The yellow, green, and red arrowed boxes represent *cI*, IS*Kpn14*, and *tet*(A), respectively. The gray and orange circles represent pBACpAK and p50825042, respectively.

Comparing the sequences of Tn*7359* from E. coli MDS-50825040-TC-Tet-3-1 and plasmid p2 from K. pneumoniae showed that plasmid p2 contained only one copy of IS*Kpn14*. The Tn*7359* composite transposon would therefore form via transposition into pBACpAK. IS*Kpn14* is an IS*1* family IS element, which can transpose through both conservative transposition and replicative transposition (Fig. 5) (41, 42). An insertion of Tn*7359* on pBACpAK could occur as a result of a replicative transposition without a resolution of the cointegrate between the conjugative plasmid and the pBACpAK vector. It could also occur as a two-step event by first inserting only IS*Kpn14* through replicative transposition, as suggested by an insertion of IS*Kpn14* in E. coli MDS-50825040-TC-Tet-2-1. The IS*Kpn14*-containing pBACpAK and IS*Kpn14*-containing conjugative plasmid could subsequently combine through a targeted conservative transposition or homologous recombination at the IS*Kpn14* of both replicons (Fig. 5).

The entrapment of Tn*7359* by pBACpAK is the first time, to our knowledge, an entrapment vector captured an entire ARG-containing conjugative plasmid. Even though the captured element was not originally a transposon, the structure of the p50825040-pBACpAK cointegrate now fits the definition of a composite transposon, consisting of two IS elements flanking a DNA fragment (21, 43). It also shows how multidrug resistance conjugative plasmids could extend their genetic complement through fusion with other plasmids, something that has recently been reported in a K. pneumoniae isolate containing the IncFIB:IncHI1B hybrid plasmid pEBSI036-1-NDM-VIR from an Egyptian hospital (44). The p50825040 conjugative plasmid from the donor would receive the *tet*(A) tetracycline resistance gene due to a fusion with the pBACpAK vector. Several studies also discovered plasmid fusion mediated by IS elements, such as IS*257*-mediated generation of multidrug resistance plasmids pSK818 and pSK697 in Streptococcus epidermis and IS*Kpn19*-mediated cointegration between plasmid pBJ114-46 and pBJ114-141 in E. coli (45, 46).

Previously, all entrapment vector studies used *cI*-*tet*(A) primers and primer walking to identify MGEs in the tetracycline resistance clones. However, we found that the *cI*-*tet*(A) region of some samples could not be amplified by both standard and long PCR protocols, so they could not be sequenced by Sanger sequencing. We therefore used WGS sequencing to characterize these clones. We found that E. coli MDS-50825040-TC-Tet-3-7 and Tet 4-38 had double insertions of IS*Kpn25* both at the *cI* gene and downstream from *tet*(A). Insertions of MGEs in other locations, not in the *cI*-*tet*(A) region, on pBACpAK were found in the WGS analysis of E. coli MDS-50627996-TC-Tet-2 as well, including an IS*26* insertion in *sopA* and a 786-bp deletion in the *cI* gene, resulting in tetracycline resistance and a failed *cI*-*tet*(A) PCR, as it lost a *cI*-*tet*(A)-F1 primer binding site. E. coli MDS-50825040-TC-Tet-2-3 showed a wild-type *cI*-*tet*(A) PCR amplicon in colony PCR screening. This plasmid was then extracted and used to represent the wild-type pBACpAK in XhoI plasmid digestion to compare with other tetracycline-resistant transconjugants. However, XhoI plasmid digestion showed that the 3-kb *cI*-*tet*(A) band of E. coli MDS-50825040-TC-Tet-2-3 shifted up to more than 10 kb (Fig. S3); it was therefore sent for WGS sequencing, which showed a 6-bp deletion in the *cI* gene, conferring tetracycline resistance, and the insertion of IS*Kpn25* at a site downstream from *tet*(A).

With the advancement of the sequencing technologies and the declining cost, using WGS sequencing to analyze all tetracycline resistance clones is becoming a viable option, as it will give information on translocation of MGEs into other locations, including the recipient chromosomal DNA. In our study, the WGS data did not show MGE insertions in the host chromosomal DNA. Such insertions are likely in a cellular population but in this case were not selected for, as our assay screened for transconjugants with a tetracycline resistance phenotype that would occur only in cells with an inactivating mutation or insertion of an MGE into the *cI* repressor gene on the pBACpAK vector.

In conclusion, we have demonstrated an approach to use the pBACpAK entrapment vector to capture MGEs from conjugative elements through a filter-mating experiment between clinical *Enterobacteriaceae* isolates and the E. coli MDS Rif Fus::pBACpAK recipient, which extended the utility of pBACpAK to other bacterial species. We also showed here that pBACpAK had the potential to capture large (120-kb) MGEs, including conjugative plasmids. Our results also demonstrated several aspects of MGE evolution after conjugation, including the rapid movement of IS elements and transposons, the formation of drug-resistance putative composite transposons, and a plasmid fusion likely mediated by IS elements.

## MATERIALS AND METHODS

### Bacterial strains and culture conditions.

All bacterial strains used in the study are listed in Table 3. All bacterial strains were grown at 37°C in Luria-Bertani (LB) medium supplemented with appropriate antibiotics (Sigma-Aldrich, UK) with concentrations as follows: chloramphenicol at 12.5 μg/mL, rifampicin at 20 μg/mL, ampicillin at 100 μg/mL, fusidic acid at 400 μg/mL, and tetracycline at 5 μg/mL.

**TABLE 3:** Bacterial strains used in this study

Strain	Characteristics and MLST^*a*^	Resistance phenotype^*b*^	Reference of source
Donor strains			
K. pneumoniae strains			
K57-33	ST461, isolated in 2009	Amp^r^	20
K68-18	ST147, isolated in 2010	Amp^r^ Chl^r^	20
K46-62	ST2134, isolated in 2007	Amp^r^	20
50825040	ST17, isolated in 2014	Amp^r^	20
50877064	ST37, isolated in 2014	Amp^r^	20
50675619	ST336, isolated in 2012	Amp^r^	20
50627996	ST11, isolated in 2012	Amp^r^ Chl^r^ Tet^r^	20

E. coli 50676002	ST131, isolated in 2012	Amp^r^ Tet^r^	20

Recipient strains			
E. coli strains			
MDS	Electrocompetent; reduced genome including deletion of mobile DNA		Scarab Genomics, USA
MDS::pBACpAK	E. coli MDS containing pBACpAK	Chl^r^	This study
MDS Rif::pBACpAK	E. coli MDS::pBACpAK with spontaneous rifampicin resistance	Chl^r^ Rif^r^	This study
MDS Rif Fus::pBACpAK	E. coli MDS Rif::pBACpAK with spontaneous fusidic acid resistance; recipient for filter-mating expt	Chl^r^ Rif^r^ Fus^r^	This study

Transconjugant strains			
E. coli strains			
MDS-K57-33-TC	Transconjugant from filter mating between E. coli MDS Rif Fus::pBACpAK and K. pneumoniae K57-33	Chl^r^ Rif^r^ Fus^r^ Amp^r^	This study
MDS-K46-62-TC	Transconjugant from filter mating between E. coli MDS Rif Fus::pBACpAK and K. pneumoniae K46-62	Chl^r^ Rif^r^ Fus^r^ Amp^r^	This study
MDS-50825040-TC	Transconjugant from filter mating between E. coli MDS Rif Fus::pBACpAK and K. pneumoniae 50825040	Chl^r^ Rif^r^ Fus^r^ Amp^r^	This study
MDS-50877064-TC	Transconjugant from filter mating between E. coli MDS Rif Fus::pBACpAK and K. pneumoniae 50877064	Chl^r^ Rif^r^ Fus^r^ Amp^r^	This study
MDS-50675619-TC	Transconjugant from filter mating between E. coli MDS Rif Fus::pBACpAK and K. pneumoniae 50675619	Chl^r^ Rif^r^ Fus^r^ Amp^r^	This study
MDS-50627996-TC	Transconjugant from filter mating between E. coli MDS Rif Fus::pBACpAK and K. pneumoniae 50627996	Chl^r^ Rif^r^ Fus^r^ Amp^r^	This study
MDS-50676002-TC	Transconjugant from filter mating between E. coli MDS Rif Fus::pBACpAK and E. coli 50676002	Chl^r^ Rif^r^ Fus^r^ Amp^r^	This study

a Multilocus sequence types (MLST) were reported in a previous study (20).

b Chl^r^, chloramphenicol resistance; Rif^r^, rifampicin resistance; Fus^r^, fusidic acid resistance; Amp^r^, ampicillin resistance.

Clinical isolates in this study were selected from a carbapenemase-producing *Enterobacteriaceae* (CPE) collection at the Norwegian National Advisory Unit on Detection of Antimicrobial Resistance (20). The whole-genome sequencing (WGS) data of these isolates (BioProject accession number PRJNA295003) were used to initially screen for strains that either did not contain tetracycline resistance genes or contained tetracycline resistance genes on chromosome-derived contigs by using ResFinder and mlplasmids (47, 48). The numbers of plasmids, MGEs, and β-lactamase genes associated with plasmid-derived contigs were also determined by using Mobile Element Finder, PlasmidFinder, and mlplasmids (10, 48, 49) and were used as criteria to select 8 potential donors for the filter-mating experiments with our E. coli MDS Rif Fus::pBACpAK recipient (see below).

### Preparation of E. coli MDS Rif Fus::pBACpAK recipient strain.

For recipient cells, the E. coli MDS strain (Scarab Genomics, USA) was used, as it has been genetically modified to remove all mobile DNA and error-prone DNA polymerases (50), reducing the possibility of false positives from the transposition of MGEs from an E. coli host and *de novo* mutations within *cI* during screening.

E. coli MDS::pBACpAK was prepared by introducing a pBACpAK entrapment vector into E. coli MDS electrocompetent cells (Scarab Genomics, USA) through electroporation. Amounts of 50 μL of the electrocompetent cells and 10 ng of pBACpAK plasmid were mixed in a prechilled 1.5-mL microcentrifuge tube and transferred to a prechilled, 0.1-cm electroporation cuvette (Bio-Rad, UK). The cells were then electroporated, and 950 μL of prewarmed SOC medium (New England Biolabs, UK) was added into the cuvette. The cell mixture was transferred to a 50-mL tube and incubated in a 37°C shaker for 1 h. After the incubation, cells were grown on LB agar containing chloramphenicol and incubated overnight. The transformants were screened and checked for the presence of pBACpAK by performing *cI*-*tet*(A) PCR with *cI*-*tetA*-F1 and ERIS primers (Table S1).

To generate E. coli MDS Rif::pBACpAK, E. coli MDS::pBACpAK was subcultured in LB broth containing chloramphenicol (for selection of pBACpAK) and incubated overnight. An aliquot of 100 μL of the overnight culture was plated onto LB agar supplemented with 20 μg/mL rifampicin and incubated overnight. The colonies grown on the selective plates were subcultured onto another fresh rifampicin selective plate to confirm their rifampicin resistance phenotype. E. coli MDS Rif Fus::pBACpAK was then generated from E. coli MDS Rif::pBACpAK with the same process but with LB agar supplemented with 400 μg/mL fusidic acid. Rifampicin and fusidic acid resistance in E. coli MDS Rif Fus::pBACpAK were confirmed by PCR amplification and sequencing of genes previously shown to be responsible for the resistance phenotypes and subsequently confirmed by WGS of E. coli MDS Rif Fus::pBACpAK transconjugants.

### Filter mating between clinical isolate donors and E. coli MDS Rif Fus::pBACpAK recipient.

The frequency of spontaneous mutation of E. coli MDS Rif Fus::pBACpAK exposed to tetracycline was determined by spreading an overnight culture of E. coli MDS Rif Fus::pBACpAK on 3 LB agar plates supplemented with rifampicin, fusidic acid, chloramphenicol, and tetracycline and incubating at 37°C for 3 days.

Filter mating was performed by following the protocol described previously (51). The donors (*Enterobacteriaceae* clinical isolates) (Table 3) and the recipient (E. coli MDS Rif Fus::pBACpAK) were grown overnight in 5 mL LB broth supplemented with appropriate antibiotics in separate 50-mL tubes. Each overnight culture was subcultured into 10 mL of fresh LB broth with no antibiotics with an optical density at 600 nm (OD_600_) of 0.1 and incubated at 37°C until mid-exponential phase (OD_600_ of 0.5 to 0.6). The cells were centrifuged and resuspended in 500 μL of LB broth. The donor and recipient cells were then mixed together and 150 μL spread on a 0.45-μm-pore-size sterilized nitrocellulose filter (Sartorius, UK) on antibiotic-free LB agar plates. Control groups were also included by adding only the donor or recipient strain to filters. After 5 h, the filters were transferred into 50-mL tubes. Cells on the filters were resuspended in 1 mL of prewarmed LB broth by vortexing the tubes at high speed for 1 min. The cell suspension was spread onto plates containing LB agar supplemented with chloramphenicol, rifampicin, ampicillin, and fusidic acid (LB CRAF agar) to select for transconjugants. Ampicillin was used to select for the transfer of the β-lactamase-containing plasmid(s) to the recipient strain. The transconjugants were confirmed by subculturing on fresh selective LB CRAF agar plates and carrying out a *cI*-*tet*(A) colony PCR (*cI*-*tetA*-F1 and ERIS primers) to confirm that they were recipient cells (Table S1).

### Screening for transconjugants with insertion of MGEs within pBACpAK.

All transconjugants were subcultured into 5 mL of LB CRAF broth and incubated for 4 h in a 37°C shaker. An aliquot of 500 μL of culture was plated onto two plates of LB agar supplemented with chloramphenicol, rifampicin, ampicillin, fusidic acid, and tetracycline (LB CRAFT agar). One of them was incubated at 37°C, while the other one was incubated at room temperature. The 4-h culture was returned to the 37°C shaker overnight, and then 100 μL of the overnight culture was spread onto another two LB CRAFT agar plates and incubated at 37°C or room temperature separately. The overnight culture was also used to subculture into 5 mL of fresh LB CRAF broth and the same plating and subculture repeated for another 3 days. All plates were checked for colony growth every day for a week, and any resulting colonies were subcultured on fresh LB CRAFT agar to confirm the tetracycline resistance phenotype.

All confirmed tetracycline resistance transconjugants were screened for insertion of MGEs into the *cI*-*tet*(A) region of pBACpAK by colony PCR with *cI*-*tetA*-F1 and ERIS primers (Table S1) as described previously (15). The colony PCR was first performed with a standard PCR protocol using 2× Biomix red (Bioline, UK) with an elongation time of 3 min to amplify up to 6 kb to initially rule out clones with mutations irrelevant in this study (point mutations, deletions, and small insertions). Clones that failed to amplify using the standard PCR were repeated with Q5 high-fidelity 2× mastermix (New England Biolabs, UK) with a 10-min elongation time to amplify up to 20 kb. The amplicons with more than a 500-bp increase in the size of the *cI*-*tet*(A) amplicon compared to a wild-type *cI*-*tet*(A) amplicon (1.35 kb) were sequenced by the Sanger sequencing service from Genewiz, Germany. BLASTN, BLASTX, and ISFinder were used to compare the sequences to nucleotide, protein, and IS element databases, respectively (52, 53).

### Genetic analysis of tetracycline-resistant transconjugants.

Clones that failed to amplify a product with *cI-tetA*-F1 and ERIS primers with both standard and long colony PCR protocols were initially analyzed by extracting their plasmids and comparing their XhoI plasmid digestion pattern with the wild-type XhoI pBACpAK digestion pattern. WGS was performed by using MiSeq version 3 with 600 cycles (300-bp paired-end reads) at the Norwegian Sequencing Centre (Oslo University Hospital, Ullevål, Oslo, Norway). Genomic DNA was extracted from the bacterial pellet using the QIAcube automated station (Qiagen, Norway) following the QIAamp DNA mini-QIAcube kit protocol. DNA libraries were prepared using Nextera DNA flex tagmentation (Illumina).

The raw reads were processed with AfterQC version 0.9.7 to trim and filter low-quality reads (54), followed by *de novo* genome assembly with SPAdes 3.13.1 (55). The contigs containing pBACpAK were identified by using BLAST to compare the assembled contigs with pBACpAK sequences. Insertion of MGEs in chromosomal DNA of the E. coli recipient was checked by using breseq version 0.35.6 to map the filtered reads with the E. coli MDS reference genome (accession number AP012306) (56). The comparison of Tn*7350*-, Tn*7351*-, and Tn*7359*-containing pBACpAK sequences with their best match from BLASTN was performed with BLAST Ring Image Generator (BRIG) version 0.95 (57). The estimation of gene copy number was done by determining the number of filtered reads mapped to each gene with BWA version 0.7.17 and SAMtools version 1.11 (58, 59) and normalized by dividing the read counts by the size of each gene. The copy numbers of each gene were calculated by dividing each normalized read count by the normalized read counts of the reference genes (the chloramphenicol resistance gene and *repE* for pBACpAK and the *bla*_NDM-1_ resistance gene and *repA* for the conjugative plasmid).

### Data availability.

Novel transposons were assigned the following Tn numbers by The Transposon Registry (43): Tn*7350* (OK245453), Tn*7351* (OK245454), and Tn*7359* (accession number SAMN21542911). The WGS data were deposited at the National Center for Biotechnology Information (NCBI) with accession numbers SAMN21542910 to SAMN21542916.
