# Supplementary material for: Intracellular Transposition and Capture of Mobile Genetic Elements following Intercellular Conjugation of Multidrug Resistance Conjugative Plasmids from Clinical Enterobacteriaceae Isolates
Source: Microbiol Spectr. 2022 Jan 19;10(1):e02140-21. doi: 10.1128/spectrum.02140-21 (PMC8768599; doi:10.1128/spectrum.02140-21)
Supplement: SUPPLEMENTAL FILE 1 — Supplemental material. Download SPECTRUM02140-21_Supp_1_seq9.pdf, PDF file, 0.9 MB [file spectrum02140-21_supp_1_seq9.pdf]

Supporting Materials

Detection of Intracellular Transposition, and Capture of Multiple Mobile Genetic Elements, Following Intercellular Conjugation of Multidrug Resistance Conjugative Plasmids from Clinical Enterobacteriaceae Isolates.

Supathep Tansirichaiya[a,b,c], Richard N. Goodman[d], Xinyu Guo[d], Issra Bulgasim[d], Ørjan Samuelsen[f,g], Mohammed Al-Haroni[b,c], Adam P. Roberts[d]#

[a] Department of Microbiology, Faculty of Medicine Siriraj Hospital, Mahidol University, Bangkok, Thailand

[b] Department of Clinical Dentistry, Faculty of Health Sciences, UiT the Arctic University of Norway, Tromsø, Norway

[c] Centre for New Antimicrobial Strategies, UiT the Arctic University of Norway, Tromsø, Norway

[d] Department of Tropical Disease Biology, Liverpool School of Tropical Medicine, Pembroke Place, Liverpool, L3 5QA, UK

[e] Department of Pharmacy, UiT the Arctic University of Norway, Tromsø, Norway

[f] Norwegian National Advisory Unit on Detection of Antimicrobial Resistance, Department of Microbiology and Infection Control, University Hospital of North Norway, Tromsø, Norway.

# Address correspondence to Adam P. Roberts, Tel: +44(0)151 705 3247. E-mail: Adam.Roberts@lstmed.ac.uk

Supplementary Figure S1. Comparative analysis of the captured transposons with their best match from BlastN analysis. A.) Analysis between Tn*7350*, Tn*7351* and plasmid pK45-67VIM from *K. pneumoniae* (Accession number HF955507). B.) Analysis between Tn*7359*-containing pBACpAK (p50825040-pBACpAK cointegrate) with plasmid p2 from *K. pneumoniae* (Accession number CP009115). They were performed with the BLAST Ring Image Generator (BRIG) (1). The blue and red open arrowed boxes represent counterclockwise and clockwise coding sequences (CDSs), respectively. Note, there is actually only one copy of IS*Kpn14* in plasmid p2. The borders of all insertions are indicated with orange boxes.

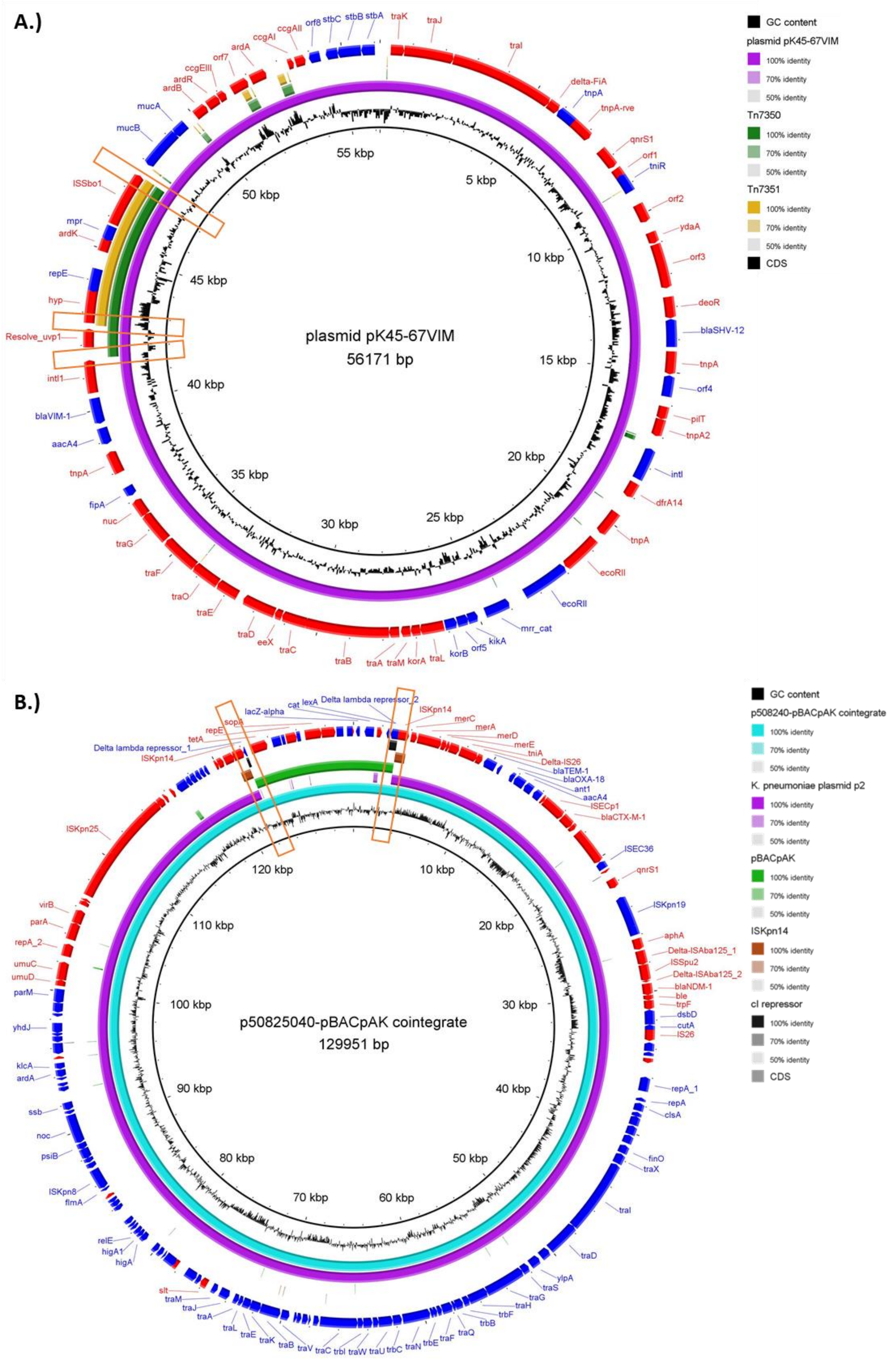
A.)
plasmid pK45-67VIM
56171 bp
55 kbp
5 kbp
10 kbp
15 kbp
20 kbp
25 kbp
30 kbp
35 kbp
40 kbp
45 kbp
50 kbp
traK
traJ
traI
delta-FiA
tnpA
tnpA-rve
qnrS1
orf1
tniR
orf2
ydaA
orf3
deoR
blaSHV-12
tnpA
orf4
pilT
tnpA2
intI
dfrA14
tnpA
ecoRII
ecoRII
mrr_cat
kikA
orf5
korB
traL
korA
traM
traA
traB
traC
eeX
traD
traE
traO
traF
traG
nuc
fipA
tnpA
aacA4
blaVIM-1
intI1
Resolve_uvp1
hyp
repE
ardK
mpr
ISSbo1
mucB
mucA
ardB
ardR
ccgEIII
orf7
ardA
ccgAI
ccgAII
orf8
stbC
stbB
stbA
GC content
plasmid pK45-67VIM
100% identity
70% identity
50% identity
Tn7350
100% identity
70% identity
50% identity
Tn7351
100% identity
70% identity
50% identity
CDS
B.)
p50825040-pBACpAK cointegrate
129951 bp
10 kbp
20 kbp
30 kbp
40 kbp
50 kbp
60 kbp
70 kbp
80 kbp
90 kbp
100 kbp
110 kbp
120 kbp
lacZ-alpha
cat
lexA
Delta lambda repressor_2
ISKpn14
merC
merA
merD
merE
tniA
Delta-IS26
blaTEM-1
blaOXA-18
ant1
aacA4
ISECp1
blaCTX-M-1
ISEC36
qnrS1
ISKpn19
aphA
Delta-ISAba125_1
ISSpu2
Delta-ISAba125_2
blaNDM-1
ble
trpF
dsbD
cutA
IS26
repA_1
repA
clsA
finO
traX
traI
traD
ylpA
traS
traG
traH
trbF
trbB
traQ
traF
trbE
traN
trbC
traU
traW
trbI
traC
traV
traB
traK
traE
traL
traA
traJ
traM
slt
higA
higA1
relE
flmA
ISKpn8
psiB
noc
ssb
ardA
klcA
yhdJ
parM
umuD
umuC
repA_2
parA
virB
ISKpn25
ISKpn14
Delta lambda repressor_1
tetA
repE
sopA
GC content
p508240-pBACpAK cointegrate
100% identity
70% identity
50% identity
K. pneumoniae plasmid p2
100% identity
70% identity
50% identity
pBACpAK
100% identity
70% identity
50% identity
ISKpn14
100% identity
70% identity
50% identity
cI repressor
100% identity
70% identity
50% identity
CDS

Supplementary Figure S2. Insertion sites of Tn*7350* and Tn*7351* in the *cI* repressor gene on pBACpAK entrapment vector. An alignment between a wild-type *cI* repressor gene and the *cI* regions from *E. coli* MDS-K46-62-TC-Tet-11 and Tet-21 containing Tn*7350* and Tn*7351*, respectively. The blue and green boxes represent Tn*7350* and Tn*7351*, respectively. The dashed arrows indicate parts of sequence alignment that were not shown in the figure. The GAAC direct and inverted repeats on Tn*7350* and Tn*7351* are indicated with purple and red boxes, respectively.

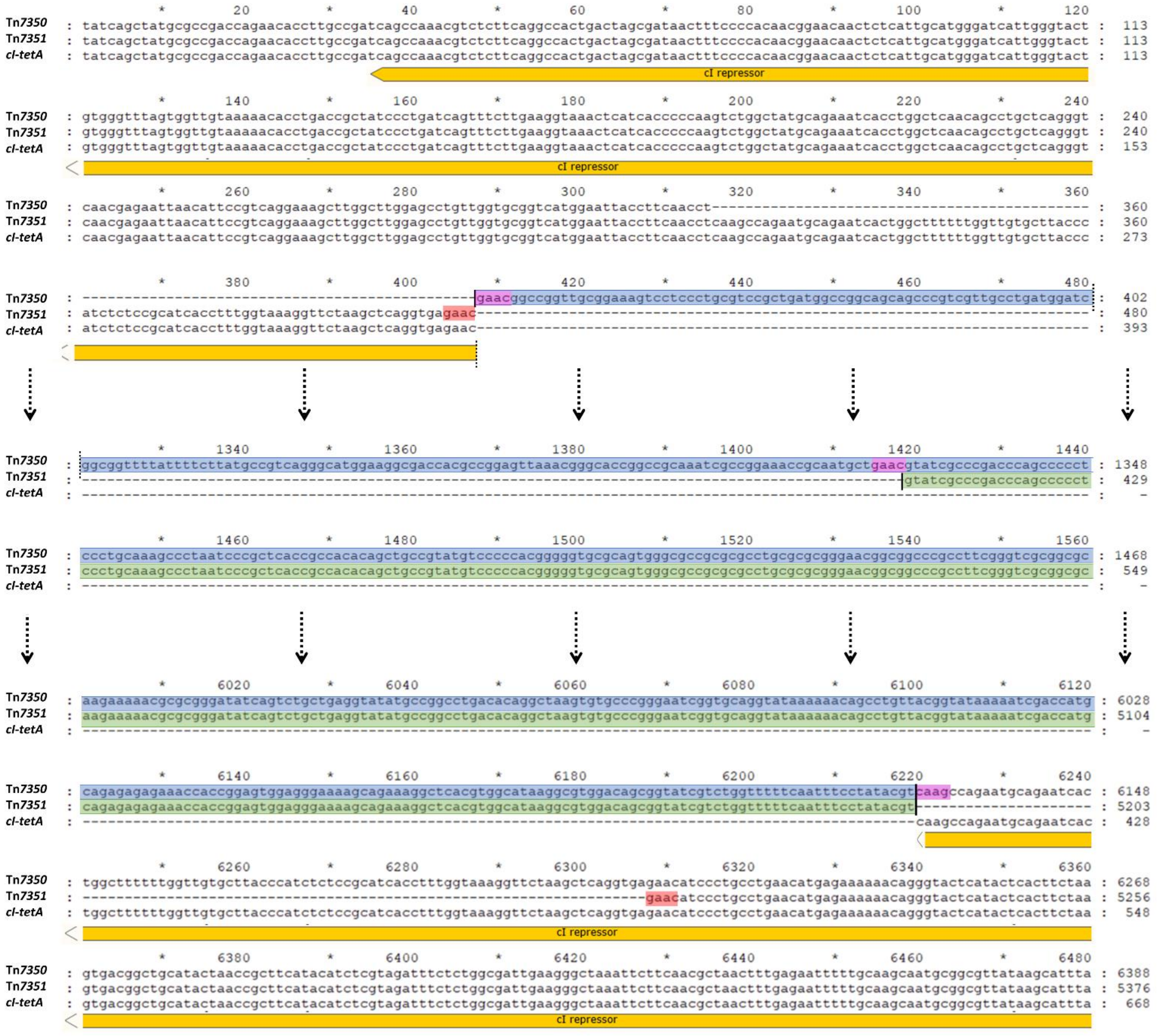

Supplementary Figure S3. XhoI digestion of plasmids extracted from *E. coli* MDS-50825040-TC-Tet-2-3, compared to the wild-type (WT). Lane M, HyperLadder™ 1kb. U: Undigested plasmid. D: Digested plasmid.

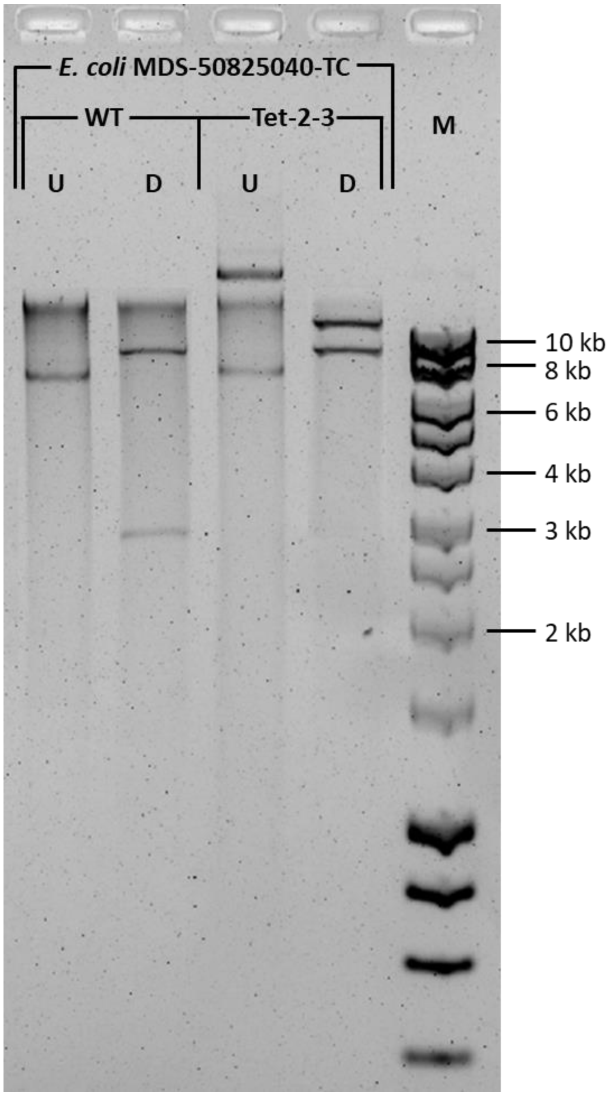

Supplementary Table S1 Primers used in this study

Primer name	Sequence (5'-3')	Gene target	Reference
**Primers to check the insertion within cI-*tet(A)* selection cartridge**			
**ERIS**	GCAAGACTGGCATGATAAGG	*cI-tetA* (reverse primer)	(2)
***cI-tetA*-F1**	CAGCCAGCAGAGAATTAAGG	*cI-tetA* (forward primer)	This study
**Primers for sequencing**			
**MKT-11-F1**	GGAGAACTGCCTGAAAGCAT	Tn*7350* extension primer	This study
**MKT-21-F1**	CGCTGTCGTGTGGAAATC	Tn*7351* extension primer	This study
**MKT-11-R1**	GATACATTCAGCCCCGCAAT	Tn*7350* and Tn*7351* extension primer	This study
**MKT-11-R2**	GTCGCAGTCAGGAAGACGAT	Tn*7350* and Tn*7351* extension primer	This study
**MKT-11-R3**	GAACAGGAGGTGGAGGAAGG	Tn*7350* and Tn*7351* extension primer	This study
**MKT-11-R4**	AACTTGTTTTCGCGGTTCTG	Tn*7350* and Tn*7351* extension primer	This study
**MKT-11-R5**	ACATTTACGGCCTGACGAAC	Tn*7350* and Tn*7351* extension primer	This study
**M5T-4-F1**	ACGAAACTGAAGCGGAGATG	IS*Sbo1* extension primer	This study
**M5T-4-R1**	CAGACGATACCGCTGTCCAC	IS*Sbo1* extension primer	This study

Supplementary Table S3 Estimation of gene copy numbers based on WGS data of tetracycline resistance *E. coli* MDS-50825040-TC

		***E. coli* MDS-50825040-TC-Strains**						
		**Tet-2-1**	**Tet-2-3**	**Tet-3-1**	**Tet-3-7**	**Tet-4-13**	**Tet-4-38**	**Tet-4-52**
**Reads**	*tet*(A)	438	404	791	578	288	540	239
	Chl^R	263	255	530	347	188	310	115
	IS*Kpn14*	538	176	1076	210	267	149	197
	IS*Kpn25*	2388	4139	4662	9035	3933	7470	3242
	bla_{NDM-1}	262	177	524	240	265	118	219
	repE(pBACpAK)	340	283	592	456	183	350	124
	*repA*_1_conjP	299	234	495	262	282	140	248
	*repA*_2_conjP	327	198	609	301	294	137	288
**Reads/ gene size**	*tet*(A)	0.363	0.335	0.656	0.479	0.239	0.448	0.198
	Chl^R	0.398	0.386	0.803	0.526	0.285	0.470	0.174
	IS*Kpn14*	0.701	0.229	1.401	0.273	0.348	0.194	0.257
	IS*Kpn25*	0.293	0.508	0.572	1.108	0.482	0.916	0.398
	bla_{NDM-1}	0.322	0.218	0.645	0.295	0.326	0.145	0.269
	*repE*_pBACpAK	0.450	0.374	0.783	0.603	0.242	0.463	0.164
	*repA*_1_conjP	0.295	0.231	0.488	0.258	0.278	0.138	0.245
	*repA*_2_conjP	0.365	0.221	0.679	0.336	0.328	0.153	0.321
**Copy number per Chl^R**	*tet*(A)/Chl^R	0.911	0.867	0.817	0.912	0.838	0.953	1.137
	Chl^R/Chl^R	1.000	1.000	1.000	1.000	1.000	1.000	1.000
	IS*Kpn14*/Chl^R	1.758	0.593	1.745	0.520	1.220	0.413	1.472
	IS*Kpn25*/Chl^R	0.735	1.314	0.712	2.108	1.693	1.950	2.282
	bla_{NDM-1}/Chl^R	0.809	0.563	0.803	0.561	1.144	0.309	1.546
	repE/Chl^R	1.129	0.969	0.975	1.147	0.850	0.986	0.941
	*repA*_1/Chl^R	0.740	0.597	0.608	0.491	0.976	0.294	1.404
	*repA*_2/Chl^R	0.915	0.571	0.845	0.638	1.151	0.325	1.843
**Copy number per bla_{NDM-1}**	*tet*(A)/bla_{NDM-1}	1.127	1.539	1.018	1.624	0.733	3.085	0.736
	Chl^R/bla_{NDM-1}	1.237	1.775	1.246	1.781	0.874	3.236	0.647
	IS*Kpn14*/bla_{NDM-1}	2.174	1.053	2.174	0.926	1.067	1.337	0.952
	IS*Kpn25*/bla_{NDM-1}	0.909	2.332	0.887	3.754	1.480	6.312	1.476
	bla_{NDM-1}/bla_{NDM-1}	1.000	1.000	1.000	1.000	1.000	1.000	1.000
	repE/bla_{NDM-1}	1.396	1.719	1.215	2.043	0.743	3.190	0.609
	*repA*_1/bla_{NDM-1}	0.915	1.060	0.757	0.875	0.853	0.951	0.908
	*repA*_2/bla_{NDM-1}	1.131	1.014	1.053	1.137	1.006	1.052	1.192
**Copy number per *repE* (pBACpAK)**	*tet*(A)/*repE*	0.808	0.895	0.838	0.795	0.987	0.967	1.208
	Chl^R/*repE*	0.886	1.032	1.025	0.872	1.177	1.015	1.062
	IS*Kpn14*/*repE*	1.558	0.612	1.789	0.453	1.436	0.419	1.564
	IS*Kpn25*/*repE*	0.651	1.356	0.730	1.837	1.993	1.979	2.424
	bla_{NDM-1}/*repE*	0.717	0.582	0.823	0.489	1.347	0.314	1.642
	repE/*repE*	1.000	1.000	1.000	1.000	1.000	1.000	1.000

		E. coli MDS-50825040-TC-Strains						
		**Tet-2-1**	**Tet-2-3**	**Tet-3-1**	**Tet-3-7**	**Tet-4-13**	**Tet-4-38**	**Tet-4-52**
	*repA*_1/*repE*	0.656	0.616	0.623	0.428	1.149	0.298	1.491
	*repA*_2/*repE*	0.811	0.590	0.867	0.556	1.354	0.330	1.957
**Copy number per *repA*_1 (Conjugative plasmid)**	*tet*(A)/*repA*_1	1.232	1.452	1.344	1.855	0.859	3.243	0.810
	Chl^R/*repA*_1	1.351	1.674	1.645	2.035	1.024	3.402	0.712
	IS*Kpn14*/*repA*_1	2.376	0.993	2.870	1.058	1.250	1.405	1.049
	IS*Kpn25*/*repA*_1	0.993	2.200	1.171	4.288	1.734	6.635	1.626
	bla_{NDM-1}/*repA*_1	1.093	0.943	1.320	1.143	1.172	1.051	1.101
	repE/*repA*_1	1.525	1.622	1.604	2.334	0.870	3.353	0.671
	*repA*_1/*repA*_1	1.000	1.000	1.000	1.000	1.000	1.000	1.000
	*repA*_2/*repA*_1	1.236	0.957	1.391	1.299	1.179	1.106	1.313
**Copy number per *repA*_2 (Conjugative plasmid)**	*tet*(A)/*repA*_2	0.996	1.518	0.966	1.428	0.729	2.932	0.617
	Chl^R/*repA*_2	1.093	1.750	1.183	1.567	0.869	3.075	0.543
	IS*Kpn14*/*repA*_2	1.922	1.038	2.064	0.815	1.061	1.270	0.799
	IS*Kpn25*/*repA*_2	0.803	2.300	0.842	3.302	1.472	5.998	1.238
	bla_{NDM-1}/*repA*_2	0.884	0.986	0.949	0.880	0.994	0.950	0.839
	repE/*repA*_2	1.234	1.696	1.153	1.798	0.739	3.031	0.511
	*repA*_1/*repA*_2	0.809	1.045	0.719	0.770	0.849	0.904	0.762
	*repA*_2/*repA*_2	1.000	1.000	1.000	1.000	1.000	1.000	1.000

References

1. Alikhan N-F, Petty NK, Ben Zakour NL, Beatson SA. 2011. BLAST Ring Image Generator (BRIG): simple prokaryote genome comparisons. BMC Genomics 12:402.
2. Bartosik D, Sochacka M, Baj J. 2003. Identification and characterization of transposable elements of *Paracoccus pantotrophus*. Journal of Bacteriology 185:3753-3763.
